# Managing a Rare Case of Mixed Extrapulmonary Small Cell Carcinoma and Adenocarcinoma of the Prostate

**DOI:** 10.7759/cureus.40701

**Published:** 2023-06-20

**Authors:** Abhinav Vyas, Sunpil Hwang, Sangeetha Isaac, Clint D Kingsley

**Affiliations:** 1 Department of Internal Medicine, North Alabama Medical Center, Florence, USA; 2 Division of Medical Oncology, National Cancer Center Singapore, Singapore, SGP; 3 Department of Oncology, North Alabama Medical Center, Florence, USA

**Keywords:** treatment-emergent neuroendocrine prostate cancer, metastatic prostate carcinoma, small cell carcinoma of the prostate, extrapulmonary small cell carcinoma, urinoma, durvalumab

## Abstract

This report presents a remarkable and unusual case of extrapulmonary small cell carcinoma (EPSCC) occurring in the prostate of a 77-year-old male patient with a previous history of prostate adenocarcinoma and multiple metastases. EPSCC is a highly aggressive form of cancer that often results in unfavorable survival outcomes, posing significant challenges in terms of management due to the absence of established treatment protocols. Despite receiving standard treatment including bicalutamide and leuprorelin, the patient’s condition showed no improvement. Consequently, the medical team made the decision to administer a carboplatin-etoposide chemotherapy regimen along with durvalumab, drawing upon the efficacy observed in similar treatment approaches for small cell carcinoma of the lung. This case highlights the critical need for further research and clinical trials to establish optimal treatment strategies for EPSCC affecting the prostate. By enhancing our understanding of this rare malignancy, we can potentially improve patient outcomes and develop targeted therapies tailored to its aggressive nature.

## Introduction

Small cell carcinomas (SCCs) are aggressive neoplasms commonly associated with a pulmonary origin. However, rare extrapulmonary SCC (EPSCC) has been reported in various sites, with an incidence in North America approximated to be 0.1% to 0.4% [1]. Among these sites, approximately 10% of EPSCC cases occur in the prostate and are associated with poor survival, with a median survival of 10 months. The rarity of prostatic SCC results in the unavailability of a formal treatment protocol. Initially described in 1977, it remained infrequently discussed in the medical literature. Current treatment is based on studies of pulmonary SCC using a combined chemo-radiotherapeutic approach with radical prostatectomy as an adjunct in only a few selected cases [2,3]. Extensive documentation exists regarding the use of chemotherapy as the primary treatment for SCC of the prostate. It has been found that etoposide/cisplatin regimens, initially used to treat SCC of the lung, exhibit remarkable effectiveness. Here, we present the case of a patient diagnosed with SCC of the prostate who is currently being treated with carboplatin, etoposide, and durvalumab.

## Case presentation

A 77-year-old male patient with a medical history of insulin-dependent type 2 diabetes mellitus, hypertension, chronic kidney disease 3a, and prostate adenocarcinoma on treatment with bicalutamide and leuprorelin presented to the emergency department with painless hematuria and lower abdominal pain. The patient used a chronic indwelling Foley catheter due to nocturia, which was replaced one week before the visit due to occlusion. The patient did not report any recent traumatic incidents. There was no fever, chills, vomiting, lightheadedness, or dizziness. The patient had undergone a prostate biopsy two months ago which confirmed infiltrating adenocarcinoma in the right and left lobes of the prostate, with Gleason scores of 3+5 = 8 and 4+5 = 9, respectively. Furthermore, the biopsy results also indicated infiltrating high-grade carcinoma with neuroendocrine features, including synaptophysin, which suggested SCC. He was subsequently started on bicalutamide and leuprorelin.

Upon presentation, the patient’s blood pressure was 123/79 mmHg, heart rate was 100 beats per minute (bpm), respiratory rate was 15 bpm, temperature was 98.3°F, and oxygen saturation was 94% on room air. He was well-developed, well-nourished, and alert on physical examination, with mild acute distress secondary to abdominal pain. The cardiopulmonary examination was unremarkable. The abdomen was soft, and there was mild tenderness in the lower abdomen; no bruit or organomegaly could be appreciated. Neurological and psychiatric examinations were unremarkable.

The initial laboratory findings showed mild anemia, with a hemoglobin level of 12.3 g/dL (normal range (NR) = 14.0-16.0), an increased white blood cell count of 17,200 cells per microliter of blood (NR = 14.3-11.0), and platelet count of 507 platelets per microliter of blood (NR = 150-450). Furthermore, the creatinine and blood urea nitrogen levels were elevated at 63 mg/dL (NR = 4-22) and 3.0 mg/dL (NR = 0.6-1.3), respectively. Urinalysis was positive for many microscopic white and red blood cells with 1+ leukocyte esterase. The liver function test was within normal limits. Hemoglobin A1c was 11.5% (NR = <5.7), representing uncontrolled diabetes mellitus. Urinalysis was positive for many microscopic white and red blood cells with 1+ leukocyte esterase. Prostate-specific antigen (PSA) was 5.21 ng/mL (NR = 4.5-5.5, age-adjusted). The levels of lactate dehydrogenase were within the normal range. Table 1 presents the lab values of the patient on presentation.

**Table 1:** Lab values of the patient on presentation.

Lab values	Reported value	Reference value range
Hemoglobin	12.3 g/dL	14.0–18.0
Hematocrit	38.9%	40.0–54.0
Mean corpuscular volume	88.7 fL	80.0–94.0
Platelet count	507 × 10^3^/µL	150–375
Blood urea nitrogen	63 mg/dL	4–22
Creatinine	3.0 mg/dL	0.6–1.3
Total bilirubin	0.7 mg/dL	0–1.0
Lactate dehydrogenase	545 U/L	313–618
Hemoglobin A1c	11.5%	<5.7
Prostate-specific antigen	5.2 ng/mL	4.5–5.5

**Figure 1:**
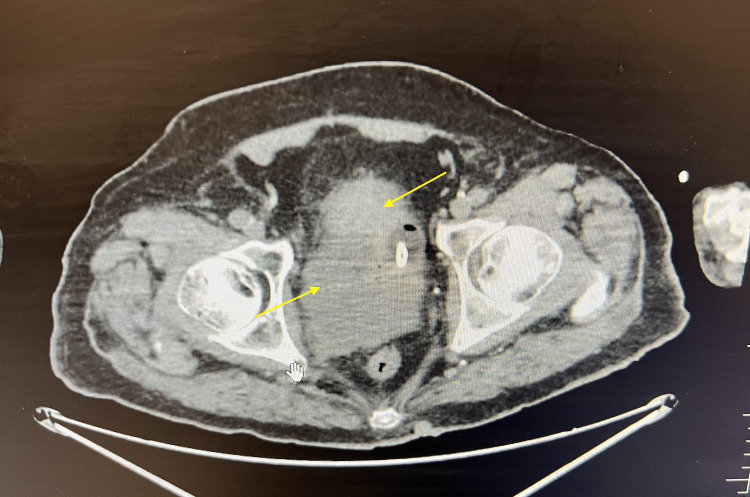
Yellow arrows demonstrate an enlarged prostate gland with heterogenous attenuation consistent with prostate cancer.

**Figure 2:**
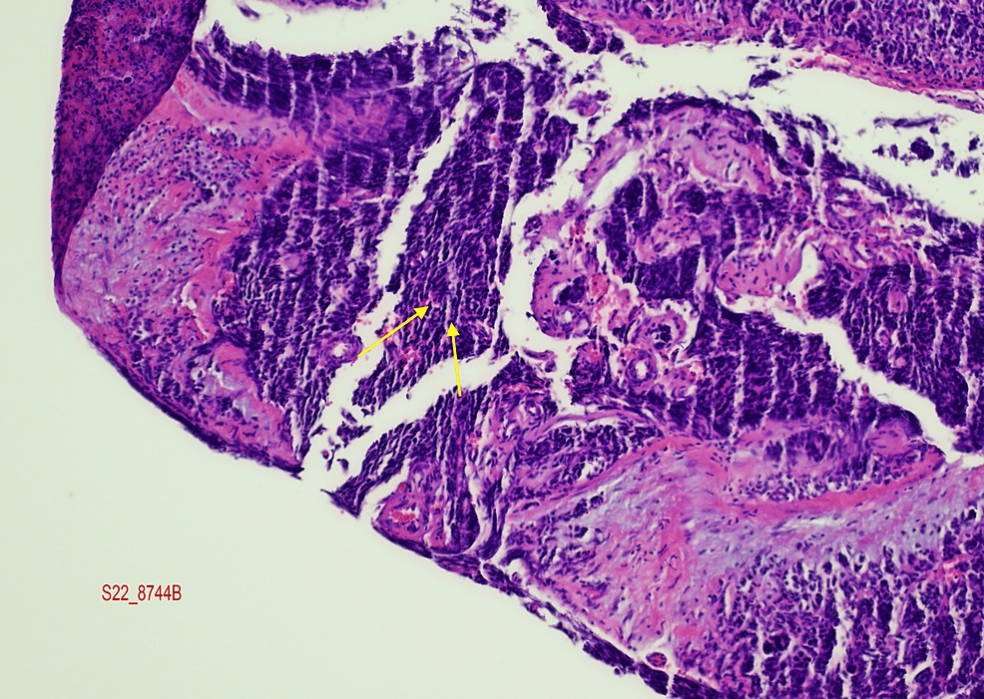
Yellow arrows demonstrate small cell neuroendocrine carcinoma cells.

Based on the initial CT scan of the chest, abdomen, and pelvis performed two months ago, the patient’s imaging results indicated the presence of several significant findings. Multiple non-calcified bilateral pulmonary nodules, measuring up to 1.0 cm, were observed, along with external iliac/pelvic lymphadenopathy. Additionally, an enlarged prostate gland with heterogenous attenuation consistent with prostate cancer was noted. The loss of fat between the prostate gland, urinary bladder, and seminal vesicles suggested the involvement of these structures (Figure 1). Subsequently, the patient underwent transurethral resection of the prostate, and the histopathology specimen revealed infiltrating prostate adenocarcinoma with a Gleason score of 4+5 = 9. Notably, the repeat pathology report indicated the presence of small cell neuroendocrine carcinoma involving 80% of the tissue, significantly increased compared to the biopsy conducted two months prior (Figure 2).

To further assess the extent of metastasis, additional imaging studies were performed. First, a non-contrast MRI scan of the brain was conducted, which showed metastatic osseous disease specifically affecting the right parietal bone. However, no definitive evidence of intracranial metastatic disease was observed on the MRI scan, suggesting that the cancer had not yet spread to the brain. Furthermore, a positron emission tomography (PET) scan was performed, revealing increased uptake at various sites in the body. Specifically, elevated radiotracer uptake was observed at C3, the sternum, the proximal left humerus, and the left lobe of the liver. However, no definitive radiographic mass was identified at these locations. The PET scan also revealed the presence of a 2.6 × 1.7 cm right-sided common iliac lymph node compressing the adjacent right ureter, resulting in right-sided hydronephrosis. Additionally, a 3.9 × 3.6 cm external iliac lymph node on the right side and an enlarged prostate gland with an invasion of the base of the urinary bladder were noted.

**Figure 3:**
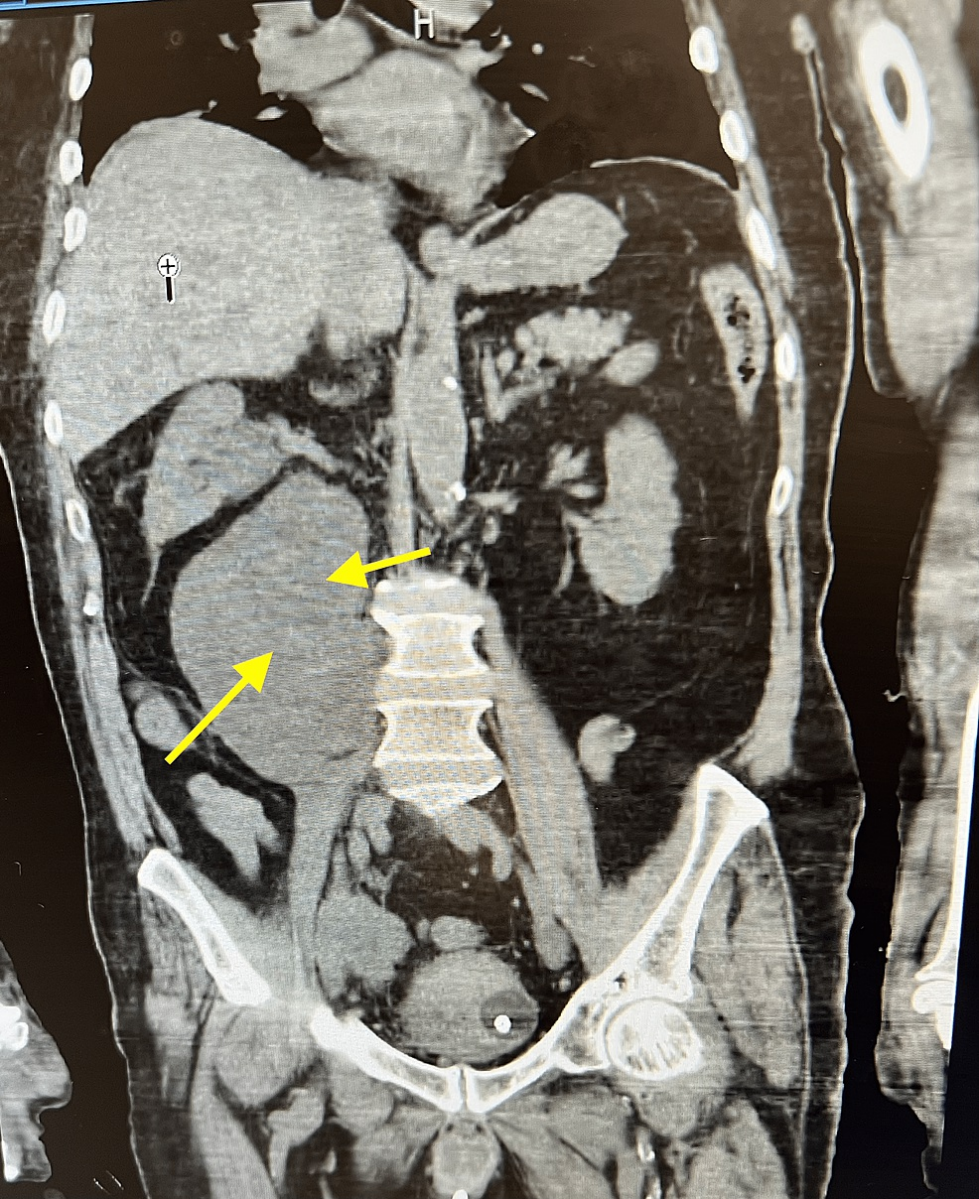
Yellow arrows demonstrate right-sided hydroureteronephrosis and an extensive retroperitoneal fluid collection posterior to the right kidney measuring 14.7 × 9.5 × 12.7 cm, consistent with urinoma.

An abdominal and pelvic CT scan was ordered for further evaluation during the current hospitalization. This scan revealed right-sided hydroureteronephrosis and an extensive retroperitoneal fluid collection posterior to the right kidney, measuring 14.7 × 9.5 × 12.7 cm, consistent with urinoma (Figure 3). Furthermore, multiple bilateral pulmonary nodules, measuring up to 14 mm, and extensive low attenuating masses throughout the hepatic parenchyma indicative of metastatic cancer were observed. Multiple enlarged pelvic lymph nodes were also identified, measuring up to 4.1 × 4.6 cm on the right side and 2.2 × 2.0 cm on the left. Further imaging in the form of thoracic and lumbar MRI revealed extensive metastasis throughout the thoracic and lumbar spine. These findings collectively demonstrated the advanced stage of cancer and the widespread metastatic involvement beyond the initial prostate tumor.

The patient initially received broad-spectrum antibiotic coverage with piperacillin-tazobactam as a treatment for obstructive nephropathy, which was subsequently de-escalated to oral levofloxacin following the results of a urine culture that revealed the growth of pan-sensitive *Klebsiella oxytoca* and *Enterococcus faecalis*. Cystoscopy was performed to further evaluate the hydroureteronephrosis, which revealed complete obstruction of the right ureter due to malignancy. Given the impracticability of inserting a ureteral stent, the patient underwent a nephrostomy to drain the urinoma. In addition, needle aspiration of the urinoma was performed to exclude the presence of malignant cells. Three days later, a nephrostogram was done to assess the right ureter, which demonstrated the resolution of urinoma with no evidence of extravasation beyond the collecting system. The patient’s hemoglobin remained stable throughout the hospitalization, and blood transfusion was not indicated.

**Figure 4:**
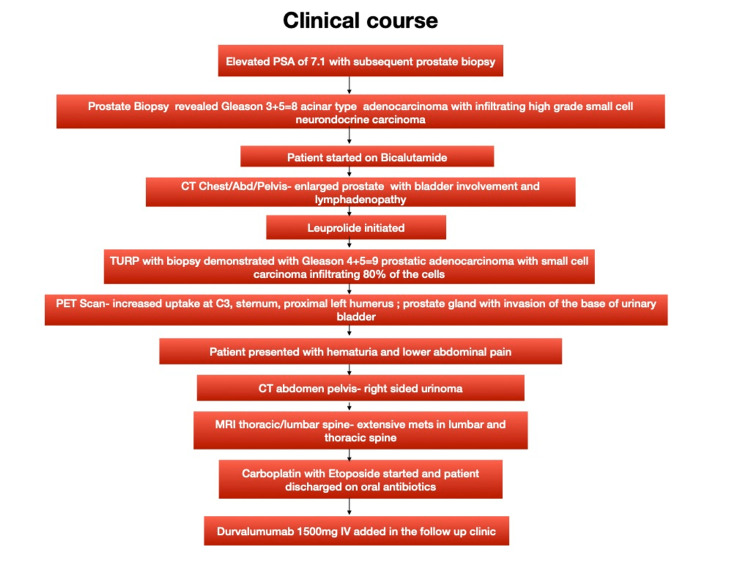
Flowchart depicting the clinical course of our patient. PSA: prostate-specific antigen; CT: computed tomography; TURP: transurethral resection of the prostate; PET: positron emission tomography; MRI: magnetic resonance imaging; IV: intravenous

Meanwhile, the patient’s chemotherapy regimen was adjusted. Considering the biopsy results and the tumor progression with transdifferentiation despite being on bicalutamide and leuprorelin, a collective decision was made to initiate a carboplatin-etoposide regimen after placing the port-a-cath in the left anterior chest wall. The patient was discharged on oral levofloxacin for three days with home health services and physical therapy. He followed up at the cancer clinic as an outpatient in three weeks for his repeat cycle. During the visit, intravenous durvalumab 1,500 mg was added, to be continued once every three weeks for four cycles, followed by maintenance of the regimen. The repeat PSA level two months later was 0.112 on the follow-up visit. European cooperative oncology group (ECOG) score on the follow-up visit was 1. Figure 4 summarizes the clinical course of our patient.

## Discussion

Prostate carcinoma is one of the most common malignancies found in men. However, EPSCC is a rare and highly aggressive form of cancer that can originate from nearly any organ, including the prostate (10%). Many patients present with early dissemination of cancer or experience rapid disease progression. Given the lack of concrete evidence for treating EPSCC, current management strategies are generally similar to those for SCC of the lungs [1]. Symptoms usually include obstructive uropathy, abdominal pain, neurologic or constitutional symptoms, hydronephrosis, hematochezia, and hematuria [4]. However, in some patients with a history of adenocarcinoma of the prostate, the disease may recur as an EPSCC after hormonal therapy, like in our patient. PSA levels in the blood can vary from undetectable to high levels, with a mean level of 4.0 ng/dL [5].

According to data from previous case reports and review articles, treatment of SCC of the prostate typically includes chemotherapy with a platinum-containing doublet (cisplatin-etoposide) [5,6]. Additionally, a retrospective review done in 1992 showed that immediate chemotherapy with or without hormonal therapy for both pure SCC and mixed adenocarcinoma of the prostate resulted in more prolonged clinical remissions. While the value of testing PSA expression remains largely uncertain for SCC of the prostate, other markers, including CD56, chromogranin, synaptophysin, thyroid transcription factor 1, and CD44, show much better specificity for SCC when compared to conventional immunohistochemical markers used for adenocarcinoma [7-9].

As demonstrated in this case, chemotherapy typically involves a platinum-based regimen combined with etoposide. However, in cases where patients have locoregional EPSCC, aggressive therapy may offer the possibility of a cure. Therefore, primary care physicians and other medical professionals who receive a diagnosis of EPSCC for their patients should exercise great caution and make urgent referrals to oncologists to actively develop a treatment plan for this condition considering its highly disseminated and aggressive nature. Failure to do so may result in rapid disease progression and dire outcomes for the patient.

A recent case report has documented a complete remission of EPSCC mixed with adenocarcinoma after 36 months [10]. As a result, it is recommended that patients with clinical and pathologic characteristics of this mixed disease should receive early multimodal therapy, including chemotherapy and androgen deprivation. However, further research is required to obtain more information on this uncommon tumor type. SCC of the prostate represents an aggressive tumor histology associated with high disease-specific mortality. It is imperative to note that individuals with low serum albumin levels and elevated serum lactate dehydrogenase levels during medical treatment may have a poor prognosis. Hence, it is highly recommended to explore alternative therapies as soon as possible after implementing conventional therapy to control symptoms [11]. Our patient did not have either of the above. Therefore, for the subgroup of patients with non-metastatic disease at the time of SCC diagnosis, there may be a benefit to incorporating local therapies with systemic therapy. However, this treatment approach should be evaluated further in prospective multicenter trials.

In a recent randomized, open-label, phase 3 CASPAIN trial in 2019 done across 23 countries, patients were equally allocated to the durvalumab plus platinum-etoposide and platinum-etoposide groups for first-line treatment for extensive-stage small cell lung cancer [12]. The combination of durvalumab and platinum-etoposide showed a noteworthy improvement in overall survival, boasting a hazard ratio of 0.73. The median overall survival rate for the durvalumab and platinum-etoposide group was 13.0 months, surpassing the 10.3 months of the platinum-etoposide group. Additionally, 34% of patients in the former group were still alive at 18 months, as opposed to 25% in the latter group [12]. Unfortunately, there was no such trial for EPSCC; however, based on this trial and more literature, we decided to start our patient on durvalumab plus etoposide and carboplatin for maximal benefits.

Therapeutic resistance to androgen receptor deprivation therapy can lead to the emergence of neuroendocrine transdifferentiation of prostate cancer. It is characterized by the loss of androgen receptor signaling during transdifferentiation, resulting in resistance to androgen receptor-targeted therapy. In addition, these tumors reactivate developmental programs associated with epithelial-mesenchymal plasticity and the acquisition of stem cell-like properties. An Aurora kinase inhibitor called Alisertib was evaluated in a recent clinical trial for neuroendocrine prostate cancer; however, only a minority of patients, including some exceptional responders, responded to the treatment. These findings emphasize the aggressive nature of EPSCC and the need for further research to develop effective treatment strategies [13].

## Conclusions

EPSCC of the prostate, also known as small cell neuroendocrine prostate cancer, is an aggressive and uncommon tumor that has a high mortality rate. While chemotherapy is a well-documented treatment for metastatic SCC of the prostate, there is a lack of specific data for mixed adenocarcinoma and SCC. Therefore, early multimodal therapy with chemotherapy and androgen deprivation is recommended for patients with clinical and pathologic features of mixed disease. SCC of the prostate is a relatively uncommon occurrence, accounting for less than 1% of de novo prostate cancers. However, it can also develop due to treatment resistance in patients with castration-resistant prostate carcinoma.
